# Health benefits of policies to reduce carbon emissions

**DOI:** 10.1136/bmj.l6758

**Published:** 2020-03-30

**Authors:** James Milner, Ian Hamilton, James Woodcock, Martin Williams, Mike Davies, Paul Wilkinson, Andy Haines

**Affiliations:** 1 Department of Public Health, Environments and Society, London School of Hygiene and Tropical Medicine, London, UK; 2 Centre on Climate Change and Planetary Health, London School of Hygiene and Tropical Medicine, London, UK; 3 UCL Energy Institute, University College London, London, UK; 4 Centre for Diet and Activity Research (CEDAR), MRC Epidemiology Unit, School of Clinical Medicine, University of Cambridge, Cambridge, UK; 5 Environmental Research Group and Medical Research Council Centre for Environment and Health, King’s College London, London, UK; 6 UCL Institute for Environmental Design and Engineering, University College London, London, UK; 7 Department of Population Health, London School of Hygiene and Tropical Medicine, London, UK

## Abstract

**James Milner and colleagues** argue that carefully considered policies to lower carbon emissions can also improve health, and we should use these benefits to push for strong climate action

In June 2019 the UK legally committed to cut emissions of carbon dioxide and other greenhouse gases to net zero by 2050.^1^ To reach this target the Committee on Climate Change says that a rapid transformation in infrastructure will be required across all sectors of the economy.^2^ What is less widely appreciated is that many of the required actions can improve the health of the UK population.^3^ These ancillary effects on health are commonly referred to as co-benefits, although since not all are beneficial “co-effects” is more accurate. Co-benefits provide an additional argument for acting on climate change.

Methods and literature in this area have grown rapidly over the past 15 years, mainly focusing on reductions in environmental pollutants such as air pollution and changes in health relevant behaviours such as physical activity and diet. We summarise key evidence on the health effects of climate change mitigation policies across four sectors responsible for a large proportion of emissions: power generation, housing, land transport, and food or diet. We report on individual sectors because this is how analyses are typically reported. However, the sectors interact, and, most notably, future power generation will have implications for housing and transport. We have not considered other sectors such as shipping and aviation or potentially important policies such as taxation or pricing mechanisms because their effects on health are less well researched. 

Our analysis focuses on the UK as an example given its legal commitment, though the issues and policy environments are similar in other high income countries. The evidence comes largely from studies included in two recent systematic reviews (box 1),^4^ ^5^ some of which we conducted. We have also used data from our studies that were published after the reviews.

Box 1 **Key sources of evidence on climate change mitigation and health**

**Deng et al, 2017^4^**

Climate change mitigation may affect various outcomes in addition to health, including economic activity, ecosystems, and food security. Deng et al’s systematic review synthesised the literature on links between climate policies and different co-benefits, examining the research methods used and the areas studied.

The authors found that health effects were among the most published type of co-benefit. Most publications were at a global level and few provided local (city) analysis.

**Gao et al, 2018^5^**

Gao et al conducted a systematic review looking at the health co-benefits of climate change mitigation to help policy makers prioritise the development and implementation of climate actions.

The study identified mitigation strategies in five domains—power generation, land transport, food systems, housing, and industry and economy—that usually resulted in health benefits. A key finding was that health benefits are likely to be multiplied by comprehensive measures across different sectors.

## Health effects of mitigation

### Power generation

Decarbonising electricity production will improve health by reducing concentrations of harmful air pollutants, including fine particulate matter (PM_2.5_), nitrogen dioxide (NO_2_), and black carbon (a climate heating component of particulate matter). Changes in power generation to achieve the 80% reduction in emissions set out in the UK’s 2008 Climate Change Act are expected to result in PM_2.5_ concentrations falling by more than 40% in the UK by 2050. This would save 500 000 to 1.1 million cumulative life years by 2154 compared with a reference scenario with no future action.^6^ The air pollution benefits might be even greater if projected adverse effects from increases in burning biomass are avoided.^7^

### Housing

The main strategy for meeting climate change targets in the housing sector is to reduce energy demand for heating through improved energy efficiency. This means reducing air leakage, improving insulation, replacing windows, improving heating systems, and switching fuels. Improving home energy efficiency by reducing unwanted air leakage presents a delicate trade-off for health—it improves home warmth and protects against outdoor air pollution but can increase exposure to pollutants generated inside the home such as radon and PM_2.5_ from cooking, fires, and smoking.^8^

A modelling study of the effects of building fabric, ventilation, and fuel switching strategies in UK houses estimated annual savings of 0.6 megatonnes (Mt) of carbon dioxide and 850 disability adjusted life years (DALYs) per million population.^9^ In England, if current building regulations for ventilation are met, improved home energy efficiency could lead to 2200 quality adjusted life years (QALYs) gained per 10 000 people over 50 years.^10^

If ventilation is reduced by more than that suggested by regulations, however, the effect on health is likely to be negative overall, with more than 700 QALYs lost per 10 000 people over 50 years because of increases in indoor generated pollutants.^10^ It is therefore important that measures are carefully designed, installed, operated, and maintained.

### Land transport

A combination of approaches is required to achieve zero-carbon transport and maximise the benefits for population health. Transport policies commonly focus on cleaner (eg, electric) vehicles and increasing active travel (walking or cycling), particularly in urban areas, as well as on reducing the need to travel (eg, home working) and travel distances. Of these actions, cleaner vehicles and shorter travel distance are likely to contribute most to reducing emissions as well as benefiting air pollution and possibly noise pollution. However, electric vehicles may not reduce non-exhaust emissions of particles and other health hazards, such as injury risk.

Active travel is likely to bring the greatest health benefits through increased physical activity.^11^ Estimates for England under an optimistic scenario, in which a quarter of the population cycles regularly and there is widespread use of electric bikes, suggest all-cause mortality could fall by 11%.^12^ If people in England were as likely to cycle as those in the Netherlands (allowing for distance and hilliness) around 18% would cycle to work and 41% cycle to school.^13^

In a modelling study for England and Wales based on European best practice for walking, cycling, and reduced car use, there was a 7.6% reduction in ischaemic heart disease plus reduced stroke, dementia, diabetes, depression, and cancers.^14^ A more ambitious scenario produced a greater than 10% reduction in disease burden from ischaemic heart disease, stroke, and diabetes and large falls in road traffic injuries.^14^

### Food and agriculture

Average UK diets contain 25% too much saturated fat (largely from red meat and dairy) and less than 70% of desirable levels of fruits and vegetables compared with World Health Organization recommendations.^15^ Increases in consumption of fruits and vegetables, together with improvements in agricultural technology (eg, reduced tillage) and reductions in red meat consumption could lead to large decreases in diet related diseases such as ischaemic heart disease.^16^ Replacing half of UK meat and dairy consumption with a combination of fruits, vegetables, and cereals could reduce dietary emissions by 19% and avert roughly 37 000 premature deaths from cardiovascular disease and cancer a year.^17^ Substituting large amounts of meat and dairy in this way is, however, unlikely.

More realistically, if average UK diets met WHO nutritional guidelines, dietary emissions could be reduced by 17% and almost 7 million years of life lost prematurely would be saved over 30 years.^18^ Further modest dietary changes could reduce emissions further and benefit health, but achieving larger reductions in emissions (>40%) would require radical and probably unfeasible changes to consumption patterns. 18

## Which changes bring the most gains?

**Fig 1:**
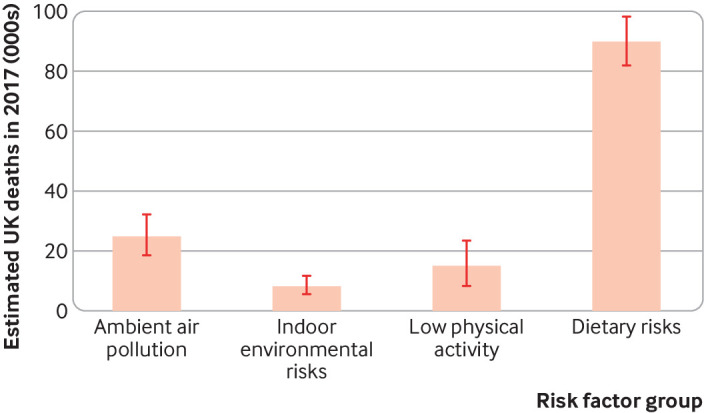
Estimated number of deaths in the UK in 2017 attributable to selected risk factors (data from the Global Burden of Disease 2017 study).^19^ Ambient air pollution includes PM_2.5_ and ozone. Indoor environmental risks include household air pollution from solid fuels, residential radon, and secondhand tobacco smoke. Bars represent upper and lower estimates

It is hard to compare the health effects of changes in different sectors. Estimates from the Global Burden of Disease (GBD) study suggest that dietary risk factors outweigh risks from air pollution, the indoor environment, and (low) physical activity in the UK (fig 1).^19^ This suggests that changing diets may offer the greatest potential for achieving public health gains. However, estimates of burdens do not necessarily reflect potential gains, and GBD estimates for physical activity are substantially lower than other published estimates, suggesting that the study may underestimate physical activity risks.^12^

An analysis of the UK economy,^20^ based on some of the studies discussed above,^9^ ^11^ ^16^ found that cleaner vehicles and increased active travel are likely to be cost effective.^20^ Benefits from agricultural technological measures combined with reduced livestock consumption are unlikely to offset the costs without further technological improvements, such as more efficient livestock farming.^20^ The benefits of home energy efficiency would be experienced over longer periods, and the interventions would breakeven once the initial costs have ended.^20^

Meeting climate change targets will, however, require action across all sectors. Some will be easier than others, with interventions that require changing the choices of millions of people, such as lower meat consumption or less driving, being potentially more difficult. 

## Making change happen

The studies described here have a clear message—that action to mitigate climate change has the potential for substantial benefits for public health in the UK. But the argument will have failed if it does not help to realise the health benefits (and avoid the disbenefits) of mitigation by translating knowledge into action—and doing so at scale and pace. Global progress on tackling climate change has been inadequate, and the rate of change remains far short of that required. Although there have been positive changes in the UK aided by strong policies in the power sector (emissions have decreased by around 44% since 1990),^21^ meeting the net-zero target will require a much faster rate of decline in emissions.^2^ Recent government decisions do not provide encouragement. For example, the weakening and withdrawal of UK housing decarbonisation policies has resulted in poor uptake of home energy efficiency measures.^22^

Cities have a vital role. Most people worldwide now live in cities, and these are responsible for over 70% of greenhouse gas emissions.^23^ Developing and implementing effective policies will require an understanding of cities as complex systems and greater priority being given to integrated approaches to sustainability and health. Urban networks such as C40 Cities, ICLEI (Local Governments for Sustainability), and the Global Covenant of Mayors are increasingly influential, and cities are beginning to respond by creating partnerships that bring different groups together to take a holistic approach at the city scale.

### Researchers

Researchers working on climate change and health can contribute in several ways. The first is to place greater emphasis on influencing policy decisions. This means not only assessing the effects of mitigation but attempting to understand how studies can contribute to decision making and tailoring the design and outputs of models accordingly. This will require working closely and sharing knowledge with policy makers, the public, and other stakeholders at all stages of the research process.

Researchers can also improve understanding of the role of public behaviour change in cutting greenhouse gas emissions and exploit the benefits of involving citizens in research. It is also important to integrate knowledge from diverse disciplines using a range of methods— for example, by working with partners from evaluative, implementation, and behavioural sciences and disciplines such as engineering, economics, and urban planning. Finally, studies show that positive messages are more likely to inspire action on climate change, and researchers should bear this in mind when reporting on mitigation studies.^24^

### Policy makers

The evidence suggests several policy actions that would contribute to reducing emissions and improving public health: decarbonising power generation, improving home energy efficiency, promoting the use of cleaner vehicles (primarily for reduced emissions), providing opportunities for walking and cycling (for health benefits), and adopting healthy and sustainable diets, particularly increased consumption of fruits and vegetables (table 1). However, policy makers must be aware of the potential for negative environmental and health effects if mitigation policies are not well designed. To avoid the adverse effects and maximise health benefits, holistic approaches to policy making will be required, looking across sectors and considering policies in combination rather than in isolation. Health should be an explicit and central component of the decision making process. The use of the Health in All Policies framework, an integrated approach to embed health into cross-sectoral decision making, in recent UK policy guidance on reducing obesity shows how this can work.^25^

**Table 1:** Summary for policy makers on climate change mitigation and health across key sectors

Main policy messages	Likely health benefits	Possible adverse effects on health
Power generation		
The UK has made progress in reducing coal use for power generation. Further progress could be achieved by increasing the supply of electricity from clean renewable sources	Improved ambient air quality reduces the harms to health	Increased use of biomass could adversely affect air quality
		Some negative environmental impacts of renewable technologies (eg, chemicals used in solar photovoltaic cells)
Housing		
Reducing energy use (and greenhouse gas emissions) from housing by improving energy efficiency. This must be done carefully, with purpose provided ventilation and particulate filters	Reduced exposure to outdoor air pollution and improved home warmth during the winter	Poor ventilation may lead to increases in indoor air pollutants, mould, etc
		Possible increased risk of overheating
		Adverse effects on mental health and psychosocial wellbeing from poorly implemented housing interventions
Land transport		
Land use policies to reduce trip distances and switching shorter journeys made by motor vehicles to active forms of travel (walking, cycling)	More active travel would have large public health benefits	Potential for increased injury risk for active travellers (pedestrians and cyclist), emphasising importance of measures to reduce road danger
Switching to cleaner fuels would reduce greenhouse gas emissions substantially	Cleaner fuels would reduce the harms from air pollution but have considerably lower health benefits than active travel	Active travellers inhale more air pollution (but impacts in UK will be small compared with physical activity benefits)
Food		
Switching to diets containing increased amounts of plant based foods and fewer animal source foods will have environmental and health benefits	Large health benefits could be achieved by increasing consumption of fruits and vegetables	Potential for increases in micronutrient deficiency
		Possible adverse environmental effects (eg, increased water use)
	Reductions in red and processed meat consumption would also have probable benefits for health in high consuming populations	Potential problems of affordability and cultural appropriateness (with implications for inequalities)

### Clinicians

As promoters of evidence based interventions, primary care providers and hospital doctors can use their influence to support climate change mitigation actions that will have benefits for health. Building and maintaining strong local community links will be vital for achieving this. In particular, doctors can recommend individual behaviour changes that promote healthier, more sustainable lifestyles, many of which can be justified through their likely benefits for health alone. These include increasing levels of physical activity and adopting healthy diets with lower environmental impacts. 

Over the longer term, the robust evidence for ancillary health benefits will enable primary care providers to advocate for ambitious climate policies at local and national level and to enact policies in their own practices that help to mitigate climate change.

**Key messages**

Action to mitigate climate change across many sectors offers an opportunity to improve public health
The benefits to health occur largely through reduced environmental pollution, increased physical activity, and improved diet
The pace and scale of change need to accelerate to meet climate change targets
Researchers need to understand how to use evidence to support action
Clinicians can use the evidence on health benefits to advocate for strong climate policies and guide their practice

## Notes

Provenance and peer review: Commissioned; externally peer reviewed.

This article is part of a series commissioned by *The BMJ*. Open access fees are paid by the Wellcome Trust, which had no involvement in external peer review, editing, or decision to publish.
